# Participatory analysis of groundnut (*Arachis hypogaea* L.) cropping system and production constraints in Burkina Faso

**DOI:** 10.1186/s13002-020-00429-6

**Published:** 2021-01-04

**Authors:** Boubacar Sinare, Amos Miningou, Baloua Nebié, John Eleblu, Ofori Kwadwo, Appolinaire Traoré, Bertin Zagre, Haile Desmae

**Affiliations:** 1 West Africa Centre for Crop Improvement (WACCI), University of Ghana (UG), PMB 30, Legon, Accra, Ghana; 2 International Crops Research Institute for the Semi-Arid Tropics (ICRISAT-WCA), BP 320 Bamako, Mali; 3 Institut National de l’Environnement et de Recherche Agricole (INERA), Ouagadougou, 04 BP 8645 04 Burkina Faso

**Keywords:** Groundnut varieties, Cropping system, Production constraints, Participatory rural appraisal

## Abstract

**Background:**

Groundnut is one of the major legume crops grown as food and cash crop across the different agroecological zones of Burkina Faso. It is ranked the 2nd important legume crop for household food, nutrition, and income generation for both rural and urban zones, contributing significantly to food supply and economy of the country. Despite its importance and breeding efforts to develop improved varieties, groundnut productivity remains low. Assessing and describing the present groundnut cropping system and production constraints as well as gender dynamics in the main production areas will help in defining the groundnut breeding priorities.

**Methods:**

A participatory rural appraisal study was conducted in three groundnut production regions (central-eastern, central-northern, and central-western). In each region, 4 villages were selected with a total of 124 farmers interviewed to collect data on socio-demographics, farming systems, cropping practices, and production constraints. Data analysis was carried out for qualitative and quantitative variables using STATA 14. Analysis of variance was conducted across regions and gender, and also between and within regions. Kendall’s coefficients were determined for qualitative variables across regions for the constraints using the pairwise rank. Pearson’s correlation was carried out to assess the relationship between variables, and the chi-square test was used to assess the difference in farmer preferences.

**Results:**

The study revealed a cropping system of groundnut in an environment largely affected by climate change and in a subsistence and extensive agriculture. There is a variation in the groundnut cropping system across the regions. Gender plays a key role in the production of the groundnut, and 48.39% of women are engaged in groundnut cropping with less access to land and production resources. A yield gap between men and women was observed with men achieving more yield than women. There was a strong correlation between the use of improved varieties and technical assistance. A strong correlation was observed between farm size and production, and farm size and sex denoting an extensive production. Production constraints, although similar, were perceived and ranked differently between regions. The lack of improved varieties, absence of agricultural credit, lack of production tools, the high price of seeds, the high price of fertilizer, drought, and disease are some of the important constraints affecting groundnut productivity.

**Conclusion:**

This study provides a recent view of groundnut cropping, allowing a good understanding of the farmers’ situation. The result will contribute to the refining of breeding priorities and guide further activities in groundnut breeding in Burkina Faso.

## Introduction

Burkina Faso is a landlocked country with an economy largely based on agriculture. Crop production is largely based on rainfall farming systems and remains vulnerable to climate hazards [1, 2]. It is in this context that over 13 million [3] people owe their food to subsistence agriculture strongly dominated by cereals, legumes, tubers, and some minor crops [4]. In west Africa, groundnut (*Arachis hypogaea L.*) plays an important role as food crop for household consumption and also as a cash crop, source of employment and incomes for smallholders in rural households [5]. In Burkina Faso, groundnut is one of the important leguminous crops widely grown thanks to its wide adaptability and dual-purpose human use and animal feed [6]. It was the number one cash crop until it was overtaken by cotton in the 1980s [7]. The groundnut production has since experienced a regression following a long drought, poor soil fertility, climate change, lack of support, and lack of promotion of the crop [8]. The country is under a highly variable spatial and temporal distribution of rainfall which is sometimes uncertain and erratic [2]. The groundnut production is characterized by low productivity and high dependence on local and inadequate production tools, coupled with a precarious environment condition. The rare increases of groundnut production observed are largely attributed to an extensive cropping system and not due to the performance of varieties [8–10].

The national groundnut breeding program at the Institut National de l’Environnement et de Recherche Agricole (INERA) remained less organized to develop improved varieties that mitigate the constraints, and hence, it had little success in improving and disseminating new high-yielding varieties. The breeding activities were limited to varietal tests of elite materials from the International Crops Research Institute for the Semi-Arid Tropics (ICRISAT) [9]. The availability of improved variety seeds and policy support remained among the challenges. There is no recent scientific study targeting the production system and constraints in the country [9]. Gender effects on production systems and also opinions of men and women on production constraints and traits preferences are reported as important factors in agriculture in many countries [11, 12]. Participatory rural appraisal (PRA) is a well-known approach and method for involving farmers and other key actors in research activities [13]. The approach allows rural people to design, share information, analyze their knowledge of life and conditions [14], take responsibility, and provide direction to the development of the new technologies. PRA is nowadays acknowledged as a strong tool in plant breeding for ensuring an efficient identification of farmer’s constraints and preferences and also helps in matching scientist criteria and farmers’ for better adoption of improved varieties. In Togo, Banla et al. [15] have conducted a PRA study as a pre-breeding activity to identify farmer’s constraints and preferences in groundnut production. Vom Brocke et al. [16] used participatory variety development as the best approach to enhance sorghum germplasm and preserve local agrobiodiversity in Burkina Faso. The current study is one of the first scientific research aiming to describe the groundnut farming system and production constraints. The result from this study will serve as a foundation for the groundnut breeding program in Burkina Faso and guide breeders in defining breeding strategies to develop high-yielding varieties responding to farmers’ needs and adapted to the local environment and market demand.

## Materials and methods

### Description of the study area

**Fig. 1:**
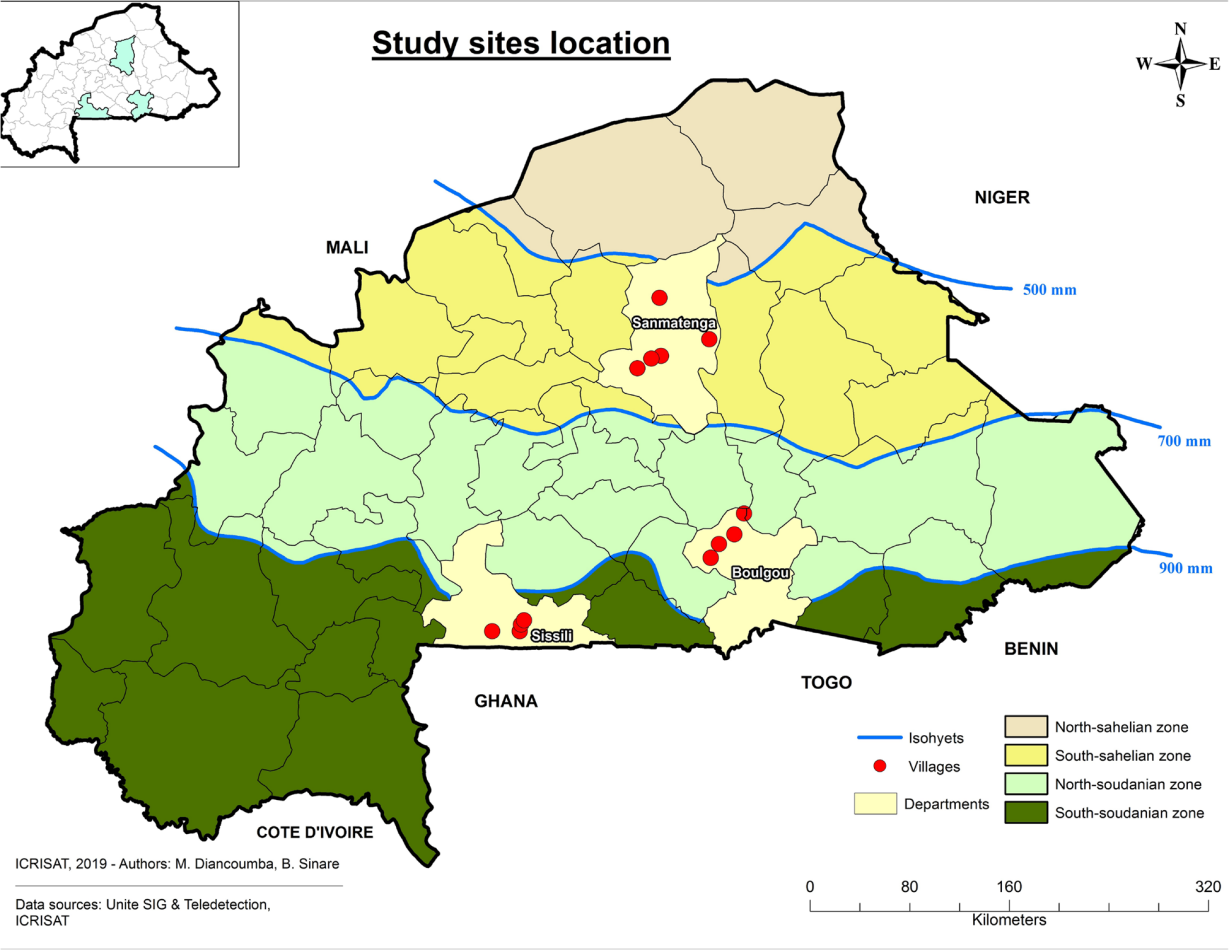
Map of Burkina Faso showing the study site, the agro-ecological zones, rainfall quantity, and Isohyets

The study was conducted by a multi-disciplinary team composed of breeders and sociologists with a good knowledge of the groundnut production areas. Local Agricultural Extension Agents (LAEA) and the Leaders of Farmers Association (LFA) were involved in this study. Questionnaire survey and focus group discussion (FGD) were used to gather information on the groundnut farming systems. Three main groundnut production regions (i.e., central-northern, central-eastern, and central-western regions) were the target areas of the study (Fig. 1). The central-northern region located in the South-Sahelian zone is characterized by annual rainfall between 500 and 700 mm and mostly sandy clay soil texture. The population is 1,202,025 people of which 53% are women [17]. The central-eastern region, located in the North-Sudanian agro-ecological zone, has an annual rainfall range of 700 to 900 mm. The population in this region is estimated at 1,132,016 people of which 53% are women [17]. The central-western region which belongs to the South-Sudanian agro-ecological zone has annual rainfall varying from 900 to 1100 mm [18, 19]. Around 1,186,566 people live in the zone, and women account for 54% [17]. Agriculture is the main activity and common to the three regions. However, numerous secondary activities are practiced by people with different frequencies from one region to another. Livestock, trading, and art are major secondary activities.

### Questionnaire design, sampling procedure, and data collection

A semi-structured survey questionnaire generated using computer package Sphinx V [20] and focus group discussion (FGD) were used to collect information in the selected areas using a multistage sampling approach. The first stage was a purposive selection of the three regions based on the importance of groundnut production reported by the Direction of National Agricultural Statistics [21]. Four villages were selected in each region based on the dynamic of groundnut farmers and platforms established by the groundnut breeding program in these regions. Groundnut farmers were then randomly sampled in the selected villages with a total of 124 farmers interviewed using the semi-structured questionnaire (Table 1). For the FGD, 3 farmers were selected among the interviewees in each village with men, women, and youth represented to assess the perceptions of each social group. One FGD was conducted in each region involving 12 farmers. Data collected from the FGD were used to support and validate the information obtained from the questionnaires. The interview questionnaire captured the socio-demographic variables (age, sex, matrimonial situation, and education level), the production system and cultural practices, groundnut farm characteristics, and agronomic variables. The FGD focused on agronomic practices, the list, and rank of the different constraints. The data were collected using the main local language, Mooré in the three regions. However, Gouroussi and Bissa languages were also concurrently used in the central-northern region and the central-eastern region, respectively. To establish easy communication, participants were divided into men group and women group during the discussion. To comply with research ethics and ensure consent, the participants were clearly informed about the purpose of the study, the kind of interview, the type of questions, and the eventual use of the information. All the participants in the individual interview and FGD gave verbal consent and voluntarily participated in the study.

**Table 1:** Study area and distribution of farmers for the individual interview and FGD

Region	Village	Geographical location		Number of farmers	
		N	W	Interview	FG
Central-eastern	Daltenga	11.95	− 0.483333	12	12
	Boussouma	11.730646	− 0.66152	7	
	Lergo	11.63352	− 0.719828	12	
	Pagou	11.79	− 0.72	6	
Total	4			37	12
Central-northern	Iryastenga	13.2329	− 1.1335	10	12
	Kalambaongo	13.211802	− 1.039553	6	
	Nessemtenga	13.010959	− 1.145495	11	
	Pissila	13.21	− 0.73	10	
Total	4			37	12
Central-western	Léo	11.1	− 2. 17	13	12
	Mouna	11.15	− 2.124262	12	
	Wan	11.184317	− 2.0713	12	
	Zooro	11.003337	− 2.084641	13	
Total	4			50	12
Total	12			124	36

*N* latitude North, *W* longitude West

### Data analysis

The collected data were coded for descriptive and comparative statistical analysis using the STATA 14 software. Analysis of variance and means were determined across regions and gender. Pearson’s correlation was carried out to assess the relationship between variables. For the FGD, the rank of the constraints in each region was used to assess the level of agreement for the ranking of the constraint among the three regions using Kendall’s W coefficient of concordance.

$$ W=\frac{12\ S}{m^2\left({n}^3-n\right)- nT};\mathrm{S}={\sum}_{i=1}^n{\left({R}_i-\overline{R}\right)}^2 $$

where *n* is the number of constraints and *m* is the number of regions. *S* is a sum-of-squares statistic over the row sums of ranks *R*_i_, and *R* is the mean of the *R* values [22, 23].

## Results

### Demographic characteristics of groundnut farmers

**Fig. 2:**
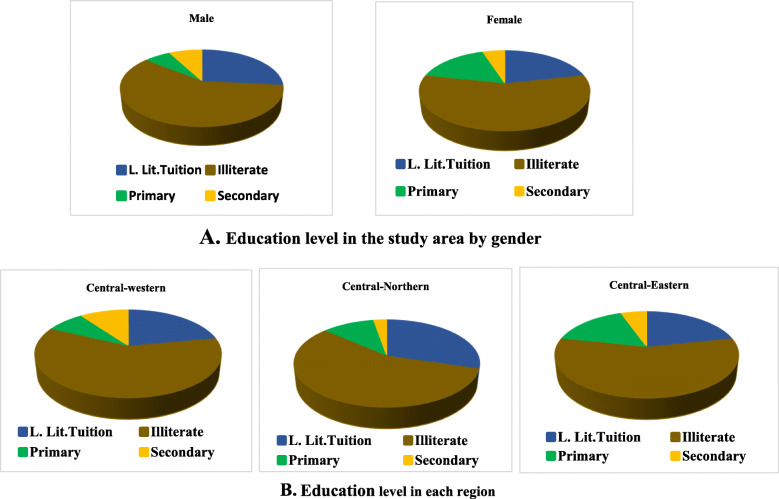
Education level in the study area and in each region

The gender distribution, matrimonial situation, literacy, and age dynamics of the groundnut farmers are given in Table 2. Among the 124 farmers interviewed, 29.84% were from the central-eastern, 29.84% from the central-northern, and 40.32% from the central-western. About 48.4% were women and 51.61% were men, suggesting a gender balance of the groundnut farmers. However, at the region level, the study revealed a big gap in gender participation in the survey. In the central-eastern region, 67.57% of participants were women and 59.46% of participants in the central-northern regions were women, while men accounted for 74% in the central-western region. In each region, both men and women produce groundnut for food as well as a cash crop. The majority of participants were married (96.77%), and the few non-married were from the central-western region (3.23%). The majority of farmers (65.32%) were between the age of 35 and 60 years; 29.03% of farmers were under 35 years while only 5.65% of respondents were more than 60 years old. More than 29% of young people were engaged in groundnut farming activities with age varying from 15 to 34. The mean age of the participants was 41 years. There was no significant difference in age across the regions, and similar proportions of each age group were observed in each region. Unlike the age of farmers across regions, there was a highly significant difference in age between men and women (*P* < 0.0001). Women farmers appear to be younger with a mean age of 37 years than men farmers (mean age of 45 years). The majority of the respondents (58.06%) were illiterate and did not attend school at all. The remaining farmers (41.94%) can read and write in either other local languages and/or the official language, French. Only 11.29% of the respondents attended primary school, 6.45% attended secondary school, and 24.20% of the participants attended local basic literacy training. Unlike the men farmers, women farmers show a higher proportion of secondary education and a low proportion of primary school education (Fig. 2). The central-northern region presents the lowest level of secondary school-educated farmers while in the central-western region, the proportion of primary and secondary school educated is almost the same (Fig. 2).

**Table 2:** Socio-demographic profile of farmers in the study areas

Region										Df	Chi-square	*P* value
Variable	Category	Central-eastern		Central-northern		Central-western		Total				
		Num	Perc	Num	Perc	Num	Perc	Num	Perc			
**Gender**	**Women**	25	67.57	22	59.46	13	26	**60**	**48.39**	2	17.3009	0.0000
	**Men**	12	32.43	15	40.54	37	74	**64**	**51.61**			
	**Total**	**37**	**29.84**	**37**	**29.84**	**50**	**40.32**	**124**				
**Matrimonial**	**Single**	0	0	0	0	4	8	**4**	**3.23**	2	6.1173	0.047
	**Married**	37	100	37	100	46	92	**120**	**96.77**			
**Age-group**	**< − 35**	9	7.26	12	9.68	15	12.1	**36**	**29.03**	4	2.69	0.61
	**35–60**	25	20.16	22	17.74	34	27.42	**81**	**65.32**			
	**> − 60**	3	2.42	3	2.42	1	0.81	**7**	**5.65**			
**Education level**	**Illiterate**	21	56.76	21	56.76	30	60	**72**	**58.06**	6	**3.8501**	**0.697**
	**Basic literacy**	8	21.62	11	29.73	11	22	**30**	**24.19**			
	**Primary school**	6	16.22	4	10.81	4	8	**14**	**11.29**			
	**Secondary school**	2	5.41	1	2.7	5	10	**8**	**6.45**

### Groundnut cropping system and practices

The cropping systems and cultural practices in the study area are summarized in Table 3. The main soil types in the study areas are sandy, clay, clay-sandy, and sandy-clay. In the central-northern region, 94.6% of farmers produce groundnut on sandy soil while in the central-western region 88% of farmers grow groundnut on clay-sandy soil. In the central-eastern region, 56.7% of interviewed farmers grow groundnut in clay or clay-sandy soil. Diverse crops are grown in the study area where on average 4 crops are grown by a producer with a range of minimum one crop and a maximum of 7 crops. On average, groundnut was ranked the second crop in terms of area and importance among the crops produced. The average ranking of groundnut between the regions was not significant. However, the average ranking of the crop by gender shows a significant difference between women and men (*P* < 0.0000). Groundnut is more important for women compared to men. It was mostly ranked the first crop produced by women, especially in the central-eastern region, while for men the crop was ranked up to 5th. About 45% of women and only 12.5% of men ranked groundnut as their first crop among the crops produced while half of the men and 38.33% of women ranked groundnut as a second crop among crops produced.

**Table 3:** Cropping systems and cultural practices in groundnut cropping in the study area

	Central-eastern	Central-northern	Central-western	Total	DF	Chi^2^	***p*** values
	%	%	%	%			
**Sowing period**							
Early of June	59.46	16.22	60	46.77	8	61.18	0.000
Middle of June	0	5.41	24	11.29			
End of June	32.43	27.03	16	24.19			
Early of July	8.11	32.43	0	12.10			
Middle of July	0	18.92	0	5.65			
**Harvest period**					6	46.29	0.0000
End of September	59.46	40.54	88	65.32			
Early of October	16.22	21.62	12	16.13			
Middle of October	2.7	32.43	0	10.48			
End of October	21.62	5.41	0	8.07			
**Soil type**							
Gravelly soil	8.11	0	0	2.42	14	66.21	0.0000
Loose soil	10.81	0	0	3.23			
Sandy soil	16.22	37.84	26	26.61			
Sandy-clay soil	8.11	56.76	62	44.35			
Clay soil	16.22	2.7	6	8.06			
Clay-sandy soil	40.54	2.7	4	14.52			
Lateritic soil	0	0	2	0.81			
**Weeding**					3	26.92	0.0000
Once	35.14	13.51	32	41.94			
Twice	64.86	86.49	68	58.06			
**Chemical fertilizer**					3	35.14	0.0000
Yes	37.84	35.14	90	41.94			
No	62.16	64.86	10	58.06			
**Organic fertilizer**					3	7.19	0.027
Yes	51.35	48.65	74	40.32			
No	48.65	51.35	26	59.68			

Early June appeared to be the most appropriate sowing period for groundnut according to 46.77% of farmers (Table 3). The mid-June and the end of June are both considered as suitable periods by 11.29% of producers. Region-wise, 60.0% and 59.5% of farmers in the central-western and the central-eastern, respectively, reported early June as a suitable period for groundnut sowing while in the central-northern the appropriate sowing period seems to be early July (32.4%) and the end of June (27.02%). Farmers generally practice groundnut weeding twice, the first at 2 weeks after sowing and the second at 30 to 45 days after sowing or at the flowering stage. More than half (65.32%) of the interviewed farmers mentioned the end of September as a suitable period for harvesting groundnut, 16.13% of farmers mentioned the early October, and 10.48% of farmers reported the mid-October. Around 59.4% in the central-eastern region as well as around 88% of farmers in the central-western region harvest groundnut at the end of September. On the other hand, in the central-northern region, 40.53% of farmers harvest at the end of September and 32.43% of farmers in mid-October.

It has been observed that groundnut is mainly cultivated in a mono-cropping system in the three regions. Intercropping is also practiced in the study area to a limited extent but mostly in the central-western region with cereals such as sorghum, millet, maize, and in some rare cases with legumes. In the study area, 11.29% of respondents rotated groundnut with other crops. About 54.3% of the respondents practiced row planting of groundnut with diverse spacing between rows and hills. The study showed that 65.32% of the farmers used chemical products for seed treatment. Although farmers use several products, the main ones are Caiman, Pacha, Thiorol, and Calthio across the three regions. It was observed that farmers in the study area use traditional practices and local knowledge for soil fertility, soil moisture, and disease management. Local practices such as composting, mixed farming, organic inputs, and crop rotation are some of the local approaches used to mitigate drought and disease, sustain soil fertility, and optimize crop yield. For example, 40.32% of the respondents in the three regions reported using organic fertilizers to mitigate drought and low soil fertility and also to increase yield (Table 3). The intercropping system is used as a measure against diseases according to farmers. In the central-northern region and central-eastern region, early sowing is a strategy for disease escape and to avoid drought. Besides, soil and water management practices such as stone and soil bunds are local cultural practices used by farmers in groundnut in the central-northern region against disease, poor soil fertility, and drought. In the central-western region, agronomic practice such as ridging “Billonnage” are well-known and more practiced compared to the other regions. Chemical fertilizer used for groundnut production is limited in the study area. Many farmers believe that there is no need to apply fertilizer for groundnut while for some of them, the reason is the lack of money. However, an important proportion of farmers (41.94%) reported applying chemical fertilizer. A large number of farmers in the central-western region (90%) reported using chemical fertilizer while 37.84% and 35.14% in central-eastern and central-northern regions reported using chemical fertilizers, respectively (Table 3) according to the producers.

### Gender implication and farm characteristics

The groundnut farm size ranged from 0.25 to 10 ha with an average of 1.072 ha (Table 4). The average production is 584.47 kg while the average yield is 681.23 kg/ha. In general, analysis of variance showed a significant difference only for the farm size (*p* < 0.0029) and pod yield (*p* < 0.0363) across the regions. The central-eastern region which presented the largest average groundnut farm size (1.57 ha) has the lowest average yield of 591.07 kg/ha. The central-western region, although presenting the smallest average farm size, differs from other regions with the highest yield of 767.15 kg/ha. All three regions possess an equal smallest farm size (0.25 ha) but the largest groundnut farm size is located in the Central-Eastern (10 ha).

**Table 4:** Groundnut farm characteristics, production, and yield in the study area

Region	Village	Groundnut farm size range			Groundnut production range			Groundnut yield range		
		Min	Mean	Max	Min	Mean	Max	Min	Mean	Max
Central-eastern	Boussouma	0.25	1.17	2	500	820	1500	500	756.66	933.33
	Daltenga	0.5	2.43	10	200	610	1000	200	648.48	1133.33
	Lergo	0.25	1.291	3	200	611.11	1200	100	408.33	1050
	Pagou	0.5	0.875	2	700	750	800	750	775	800
	**Total**	**0.25**	**1.57**_**b**_	**10**	**200**	**661.53**_**a**_	**1500**	**100**	**591.07**_**a**_	**1133.3**
Central-northern	Iryastenga	0.25	0.675	1	266.66	652	1433.33	666.66	673.33	680
	Kalambaongo	0.5	1.58	3	216.66	944.44	2266.66	433.33	634.88	850
	Nessemtenga	0.25	0.613	1	200	365.75	733.33	400	587.77	933.33
	Pissila	0.25	0.525	1	150	234.07	366.66	100	703.33	973.33
	**Total**	**0.25**	**0.76**_**a**_	**3**	**150**	**508.79**_**a**_	**2266.66**	**100**	**643.24**_**b**_	**973.33**
Central-western	Léo	0.5	0.98	1.5	216.66	769.25	1233.33	216.66	875.51	1492.06
	Mouna	0.25	0.708	1.5	143.33	425.55	123.33	433.33	751.38	1233.33
	Wan	0.5	1.125	4	236.66	557.58	983.33	473.33	1049.9	1600
	Zooro	0.5	0.903	2	216.66	593.58	1500	383.33	589.6	1000
	**Total**	**0.25**	**0.93**_**ab**_	**4**	**143.33**	**602.5**_**a**_	**1566.66**	**216.66**	**767.15**_**c**_	**1600**
**Total**		**0.25**	**1.07**	**10**	**143.33**	**584.47**	**2266.66**	**100**	**681.23**	**1600**

Means within a column followed by the same letter(s) are not significantly different

Means within a column with different letter(s) are significantly different

The study indicated a significant difference within regions for the farm size by sex although the ANOVA result showed no significant difference across regions in the study area for the farm size by sex. In each region, the smallest farms usually belong to women while the largest farms are owned by men. Only the central-western region showed gender balance for the average groundnut farm size. The analysis of variance of the average yield by gender was significant (*p* < 0.0252). The average yield obtained by men was higher than the average yield obtained by women (Table 5). A similar observation was made for the average production (*p* < 0.0000) with men production almost twice that of the women’s average production.

**Table 5:** ANOVA of groundnut farm size, production, and yield by gender

Region	Sex	Groundnut farm size		Groundnut production		Groundnut yield	
		Mean	***P*** value	Mean	***P*** value	Mean	***P*** value
**Central-eastern**	Women	1.07_a_	0.0086	572.54_a_	0.0344	502.77_a_	0.0245
	Men	2.625_b_		829.62_b_		750_b_	
**Central-northern**	Women	0.6_a_	0.345	297.46_a_	0.0006	624.5 _a_	0.52
	Men	1_a_		804.66_b_		688.73 _a_	
**Central-western**	Women	1.01_a_	0.5355	426.38 _a_	0.0536	769.44 _a_	0.97
	Men	0.89_a_		645.061 _a_		766.17_a_	
**Total**	Women	0.88_a_	0.0715	421.93_a_	0.0000	614.89_a_	0.0252
	Men	1.24_a_		724.575_b_		750.53_b_	

Means within a column followed by the same letter(s) for each region are not significantly different

Means within a column with different letters for each region are significantly different

Table 6 shows some significant correlations between farmers’ characteristics and farming system variables. Negative and significant correlations were observed for farmers’ age and the technical assistance, age and use of improved variety. A positive and significant correlation has been observed for sex and production, sex and yield, and also sex and the rank of groundnut. Groundnut production is positively correlated to field size, yield, and sowing period with high significance.

**Table 6:** Correlation among farm characteristics and farming system variables

	Age	Sex	T.A	U.I.S	E.L	F.S	Prod	Yield	Exp.	RAC	ASP	AHP
**Age**	1											
**Sex**	0.3454**	1										
**T.A**	**−** 0.1851*	0.0344	1									
**U.I.S**	**−** 0.1912*	0.0231	0.6368**	1								
**E.L**	**−** 0.2484**	**−** 0.041	**−** 0.0769	**−** 0.0415	1							
**F.S**	0.1422	0.1624	**−** 0.0434	**−** 0.1382	**−** 0.0515	1						
**Prod**	0.2556**	0.4111**	**−** 0.0602	**−** 0.1208	0.009	0.3137**	1					
**Yield**	0.0536	0.2334*	**−** 0.0831	**−** 0.1509	0.1873	0.1119	0.4065**	1				
**Exp.**	0.2625**	**−** 0.0172	**−** 0.0037	**−** 0.0878	**−** 0.0316	0.1231	**−** 0.0089	0.102	1			
**RAC**	0.1079	0.3662**	**−** 0.1953*	**−** 0.0658	0.1308	**−** 0.1568	**−** 0.0625	0.0588	0.0008	1		
**ASP**	**−** 0.1256	-0.2483**	**−** 0.2098*	0.0632	0.1761	**−** 0.2175*	**−** 0.2773**	**−** 0.1928	0.0242	0.1976*	1	
**AHP**	0.1404	0.1348	**−** 0.2455**	**−** 0.171	0.081	0.138	0.1902	**−** 0.0135	**−** 0.0299	0.1039	0.3962**	1

Signification code : ** = 0.01; * = 0.05

*T.A.* technical assistance, *U.I.S.* use of improved seed, *E.L.* education level, *F. S.* farm size, *Prod* production, *Exp* experience, *RAC* groundnut rank among crops produced, *ASP* appropriate sowing period, *AHP* appropriate harvested period

### Cultivated groundnut varieties

**Fig. 3:**
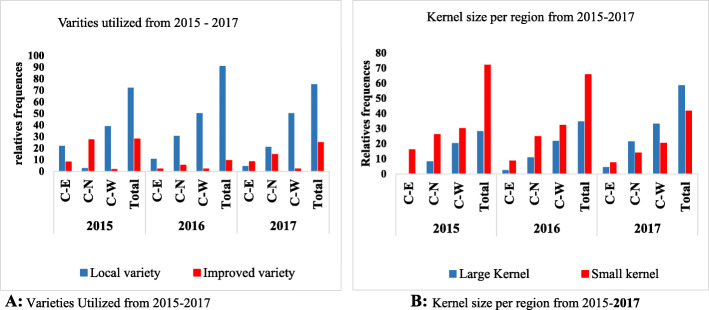
Types of varieties and characteristics of their kernel size in the study areas from 2015 to 2017. **a** Varieties utilized from 2015 to 2017. **b** Kernel size per region from 2015 to 2017. C-E, Central-Eastern; C-N, Central-Northern; C-W, Central-Western

The analysis of the type of varieties grown in the last 3 years (2015-2017) shows a large proportion of the local varieties each year and region (Fig. 3). The relative frequencies for the local varieties grown were 87.9%, 90.63%, and 75% for 2015, 2016, and 2017, respectively, against 12.1%, 9.37, and 25% of improved varieties for the same period. Most of the respondents reported the unavailability of the improved varieties. Besides, most of the varieties used in the study area are characterized by a small kernel size (Fig. 3). Indeed, 72% of the utilized varieties in 2015 were characterized by a small kernel and 28% by a large kernel. The proportions were 65.59% for the small kernel and 34.41% for the large kernel in 2016 and 58.51% for the small kernel with 41.49% for the large kernel in 2017. According to the farmers, the small kernel varieties are widely spread and easily accessible. In the study area, variety with a small kernel is often identified by farmers as a local variety which may not always be true. In general, groundnut varieties are usually identified using their seed color and or their oil content. Farmers use the local name “Nangoury peelga” or “Nangoury kaam” for varieties with red color and high oil, respectively, whether it is a local or improved variety. Thus, some local varieties are named “Nangoury peelga” or “Nangoury miougou” while others referred “Nangoury–kaam”. In the central-eastern region, names such as Soumyanga, Boanga, Kombombalga, Mayoro, Dalga (3–4 grains), and Zampou have been reported to be used by farmers for local varieties. In the central-northern region, Ballolé, Miougou, and Peelega are famous local varieties used by farmers. In the central-western region, local varieties such as Tchanabatwa, Dagarèsiè, Soudjana, Soudkounkolou, and Soudaro are used. Some improved varieties such as SH470P and Te3 are called “Nangoury peelga,” and Fleur 11 is called “Nangouri–kaam”. Specific names are also given to some improved varieties: Nafa, Miou Palé, Lokré Toinwaré (drought tolerant), Beeda (big grain), and Soukeba in the study area. Additionally, the name Nangoury changes to Sinkam depending on the region.

### Different local use of groundnut in Burkina Faso

The study revealed multipurpose use of groundnut in the study area including food, cash source, animal feed, and ecological services. Groundnut is an extremely versatile crop being used in a wide range of food products. It is used increasingly as roasted with salt commonly called “marba-tigue”. Boiled groundnut and fresh ones are also eaten daily, and its raw products are included in varying food preparation. It is even prepared mixed with caramelized sugar called “nangour-siido,” which is highly appreciated and sold everywhere in the country. The crop is also crushed after being roasted to produce groundnut butter which is used as the main ingredient in several foods. The butter is used in basic food preparation with cereals, tubers, and vegetables. For example, it is used in the preparation of local foods such as “Baag-benda or Zind-zangsenga” and “Bito-zindo” from a mixture of sorrel leaves, cowpea leaves, and millet or sorghum grains. Groundnut cake, produced after oil extraction, is used as a nutrient-rich food. In the central-northern region, groundnut cake is one of the groundnut-derived products consumed by dwellers as common snacks. The cake is also crushed and mixed with spices to marinate meat for a roast. As an animal feed, groundnut is considered as a rich fodder preferred by livestock, and the haulm is used to feed animals either fresh or dry. Rarely used in manure, after harvest, haulms are always dried and can fetch a good price for cattle, goat nutrition, and in a few cases for sheep. Additionally, groundnut is used in mixed cropping systems or rotation systems, which is a good strategy for a farmer to enhance soil fertility and prevent some crop diseases.

### Groundnut production constraints

In the FGD, farmers identified constraints and made a ranking of the constraints (Table 7). The constraints listed by farmers in FGD were similar from one region to another region. But there was no concordance of the ranking of the constraints across the regions. Each constraint was perceived with different ranks from one region to another region. For example, farmers in the central-eastern region ranked soil pest first while the short period of rainfall and drought were equally ranked first in the central-northern region. The problem of land ownership was reported as the number one constraint in the central-western region. In general, the main constraints included lack of improved varieties, lack of production tools, the high price of seed, low-yielding varieties, pest attacks, diseases, and drought.

**Table 7:** Groundnut constraint rank in each region and across the region using FGD

Groundnut production constraint	Constraint rank			
	Central-eastern	Central-northern	Central-western	Mean
Lack of improved seed	4	4	2	**4.5**
Lack of short-maturity varieties	3	3	5	**4.5**
Short period of rainfall	2	1	3	**2.17**
High price of improved seed	6	6	11	**4.67**
Diseases	4	5	4	**5.5**
Drought	3	1	8	**7.67**
Pest	1	8	10	**7.67**
Lack of training	5	2	12	**7.83**
Problem of land ownership	8	9	1	**8.33**
Lack materials	9	3	6	**8.33**
Soil poverty	7	7	7	**9**
Lack of reliable market	6	8	5	**9.5**
Problem of conservation	10	8	9	**11.33**
**Kendall’s W**	**0.431**			
**Chi-square**	**15.51**			
**F distribution** ***p*** **value**	**0.214**			

**Fig. 4:**
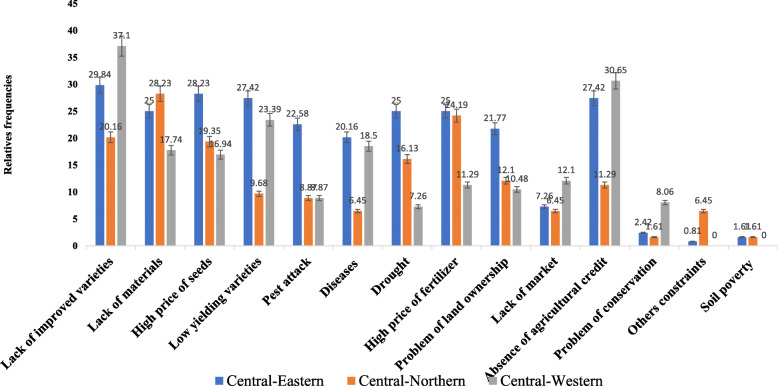
Groundnut production constraints in each region

The importance of the production constraints obtained from the individual questionnaire in each region is presented on Fig. 4. The analysis of the constraints in each region reveals that each constraint is perceived differently from one region to another as was observed in the FGD.

In the central-northern region, lack of materials (production tools), the high price of fertilizer, and lack of improved varieties were frequently reported while lack of improved varieties, the high price of seeds, and low-yielding varieties appeared frequently as main constraints in the central-eastern region. In the central-western region, lack of improved varieties, absence of agricultural credit, and low-yielding varieties were considered the top 3 production constraints. Across the regions, in general, the lack of improved varieties, the lack of materials (production tools), absence of agricultural credit, the high price of the seed, and the low-yielding varieties appear to be the top five important constraints with 13.79%, 11.23%, 10.98%, 10.22%, and 9.58% of respondents ranking of the constraints, respectively. These were closely followed by the high price of fertilizer, drought, the problem of land ownership, pest attack, and diseases.

## Discussion

### The groundnut cropping system

The present study revealed a subsistence groundnut farming system dominated by smallholder farmers in Burkina Faso. Groundnut cropping is characterized by extensive and low external input with significant variability in production practices across regions, largely depending on the rainfall which follows a north-south gradient, soil types, and socio-economic conditions. Farming systems in west Africa and especially in Burkina Faso were characterized by huge variability from one region to another with poor technology and low investments [2, 24, 25] including the groundnut production system [2, 9]. Nikiema [26] reported a varying onset of rainfall for the different agro-climatic zones in Burkina Faso. The early sowing period of groundnut observed in the central-western and central-eastern regions compared to the central-northern region could be related to the onset of effective rainfall in June giving farmers 4 to 5 months growing period. In these regions, early sowing in a long duration growing season allows farmers to cope with drought issues. This practice is coupled with the use of short-duration groundnut varieties for an early harvest. A strategy for some farmers especially in the central-western region is to plant a second crop such as yam, maize, cowpea, okra, and sorrel enabling them to produce two crops in one season and thereby increasing income. In the central-northern region, farmers experience an intermittent start of rain with often dry spells after the first rain [27] which results in the widespread of the sowing period faced by farmers. This situation often forces farmers to wait for effective rain to plant to avoid crop failures. In Burkina Faso, groundnut is produced with rudimentary farming practices and techniques [10]. The current study also revealed poor cultural practices where production is mainly carried out in monoculture associated with relatively low use of organic and chemical fertilizers due to limited availability of the fertilizer and the finance to acquire it. In some areas, for example, in the central-northern and central-eastern regions, fertilizer is used with improved varieties coupled with soil and water conservation techniques to cope with poor soil fertility. Such practice has been reported as an effective method to restore soil fertility and increase productivity [28, 29]. An increase of crop farmland using fertilizer was reported from 7% in 1993 to 30% in 2006 [30] in Burkina Faso, but the average fertilizer rate was reported low, 11 or 12 kg/ha [31, 32]. The high proportion of farmers using fertilizer in the central-western region could be attributed to several factors such as input subsidy, the agricultural potential of the area, and access to agricultural extension services. Cotton and maize are important crops in the region where farmers apply fertilizer and inputs. The fertilizer farmers receive for cotton or maize is often shared with the other crops and fields [33]. Therefore, although a large number of farmers reported using fertilizer, they do not apply the recommended amount of fertilizer.

Gender, age, and education level have significant implications in groundnut farming in Burkina Faso. The high number of women involved in groundnut production, especially in the central-northern and central-eastern regions, may be explained by their importance at each level of the groundnut value chain (production, trading, and processing). Groundnut processing represents a primary cash source for women as one of the principal activities of women in the offseason, especially in the central-northern region. Women were reported to play an important role in groundnut production, trading, and processing in West Africa [11, 34–37]. The groundnut average farm size differs between regions (*P* < 0.0029) with the central-eastern region having the largest farm size. The ANOVA for the average yield was significant across the regions (*P* < 0.0363), and the central-western region presents the highest average yield. It has been reported that the central-eastern region accounts for the largest area under groundnut cropping in Burkina Faso [8, 38]. Yet, this region seems to have the lowest average yield.

In the study area, although women are in numerical importance among groundnut farmers in the central-eastern and central-northern regions, they possess small farm size, almost 50% smaller than men farm size. Women farmers in the drought-prone areas of sub-Saharan Africa and South Asia were reported to have limited access to major productive resources, organic fertilizers [39–41]. Similarly, women’s access to land and production resources such as labor remains one of the challenging characteristics of agriculture in West Africa, especially in Burkina Faso [42, 43]. The lack of financial means, labor, and access to land are some of the reasons justifying the differences in farm size between men and women. Besides, land accessibility to women is limited in those regions because of socio-cultural factors (inheritance by lineage, religion, social hierarchy, decision-making power, etc.) and traditional common property systems. These impact women’s empowerment. Indeed, in Burkina Faso, land ownership and access, access to natural resources such as soil and water, are largely governed by men and land ownership is exclusively inherited through the family lineage from father to son [44]. Yet in the Central-Western region, some women’s groundnut farm sizes are as big as men’s farm sizes. These women are mostly groundnut traders who grow groundnut for sale. It has been reported that the proximity of the zone with Ghana offers a more interesting market for the sale of groundnut [8, 10]. So, these women do not hesitate to invest and often rent or buy land for groundnut production. Better access to land may also be explained by the social structure in this region which is more flexible and open, giving more access to land by women.

The average production across the regions did not show a significant difference, which is characterized by low productivity. However, there is a significant difference in average production and average yield between men and women with men achieving higher average yield. This result reflects the gender implication in groundnut production in Burkina Faso. Recent research on the implication of household models on agriculture production in Burkina Faso reported similar observations [42]. The significant and positive correlation between the sex and the yield and also between the production and the farm size not only confirm the gender effect on groundnut production but also underline the dependence of groundnut production to the farm size, a manifestation of an extensive cropping system. This extensive farming system has been highlighted by [45]. It should be noted that a large gender difference in farm size and yield does not imply that neither women are less efficient in groundnut cropping than men, nor more access to land by women will increase the production or the yield. But the yield gap between men and women is imputable to several constraints such as access to inputs, training, finance, and marginal land, which are associated with women farmers’ conditions in West Africa. Monyo and Varshney [41] reported a similar observation in sub-Saharan Africa and South Asia where women farmers still have limited access to major productive resources.

### Challenges and constraints in groundnut production

The low productivity of groundnut observed in the study area is attributed to diverse production constraints. In general, the lack of improved varieties, absence of agricultural credit, lack of materials (production tools), the high price of seeds, the high price of fertilizer, drought, and disease are some of the major yield-limiting factors compromising groundnut production in Burkina Faso. Similar constraints were previously reported on groundnut [9, 46–48]. Although the same constraints were listed across the study area, the constraints were ranked differently from one region to another. The central-eastern region and especially the central-northern region have a growing season known to be shorter [49]^,^ and farmers reported increasingly the unpredictable and unreliable rainfall distributions which can justify the rank of drought, short period of rainfall, and lack of short-maturity varieties ahead in these regions. To mitigate the constraints, some short-duration (90 days) varieties (e.g., TE3, TS-32-1, CN 94) have been introduced and promoted by INERA [50]. The current study revealed that there is limited use of improved varieties in the study area with the lowest use in the Central-Western region. According to most respondents, the reasons are the lack of improved varieties, the inaccessibility and unavailability of the improved varieties and the lack of money to purchase seeds. Monyo and Varshney [41] reported such factors are hampering the use of improved varieties in West Africa. Some respondents mentioned the lack of groundnut seed production companies, making access to seed very difficult. The abandonment of the groundnut sector for the benefit of other cash crops (e.g., cotton, sesame) by authorities and the limited policy support in groundnut breeding may be the causes of the disorganization of the groundnut seed production system [51, 52]. Some farmers do not even know where and how to get the improved varieties. However, it should be noted that the study did not have a reliable identification system of the varieties used by the farmers. As a result, a producer could have used an improved variety that he/she has considered as a local variety. Some farmers restrict themselves from buying the improved seed in the belief that the improved seed must come from the Local Agriculture Extension Agent (LAEA) or NGOs or groundnut platform members. Indeed, this is reflected in the strong positive and significant correlation of the technical assistance and the use of improved varieties. For example, a relatively higher proportion of improved varieties used was observed in the Central-Eastern and Central-Northern region where ICRISAT has reported an increase of community-based improved groundnut seed production and marketing under the Tropical Legumes project [41].

Young farmers are the largest users of improved seed which is confirmed by the significant negative correlation of age of farmer and the use of improved varieties. This could be because the young farmers are more educated compared to the elders, which gives them better access to information and makes them more open to new technologies. The low level of education observed in the study areas suggests the need to use local languages for the dissemination of new improved varieties of groundnut. This will facilitate awareness creation and the uptake of the new varieties for their rapid adoption. The educated farmers can serve as communicators about the potential of new varieties or other technologies: those farmers with primary and secondary level may be useful in gathering information regarding groundnut farming. They can also serve as facilitators when introducing new technologies in groundnut farming communities in the study areas. However, the important proportion of local literature tuition farmers could be helpful to seed companies for the promotion of new varieties if the groundnut seed system was well operating.

Another noticeable fact revealed by this study was the higher proportion of the small kernel groundnut used in the area. The central-western and central-northern regions present a high proportion of small kernel groundnut used. For the respondents, the small kernel varieties of groundnut are widely spread and used because farmers have limited choice for large seed. Farmers reported that the limited choice in terms of varieties forces them to rely on the local varieties and the small kernel varieties which are less productive and even not preferred. Formerly considered as a cash crop because of oil extraction, varieties with small kernel were promoted because of their high oil content and are thus the most available groundnut varieties disseminated in Burkina. Although farmers remained unsatisfied with yield potential, their complaints about the inaccessibility of these varieties prove the value of small kernel varieties. Involving farmers in breeding activities to choose their preferred varieties and reinforce their capacities on seed production could help to make the new varieties available and accessible.

Farmers mentioned foliar diseases such as rosette disease, leaf spots, and rust as important constraints. In the Central-Western region, diseases were reported with predominant occurrence causing significant loss of production. This may be attributed to the high level of humidity due to good annual rainfall exceeding 1000 mm [26] favoring disease development. Fungal and viral diseases were reported as yield-limiting factors for groundnut in West Africa [15, 46, 47]. Additionally, farmers mentioned post-harvest loss due to storage pests. A large proportion of the harvest is often sold at a low price to avoid the problem of storage loss. Farmers reported that groundnut fetches a better price when it is sold fresh in pods. Another important issue is that farmers sometimes may leave groundnut in the field after harvest and/or the harvest is postponed due to priorities given to farm activities for stable crops such as sorghum and millet. This practice causes a lot of damages and losses due to termite damage or pod loss from peg breakage due to the drying of soil at harvest time. It should be noted that combinations of cultural practices such as intercropping and soil fertility management are used by farmers in the study area as strategies against diseases. However, local practices of disease management generally remain limited as farmers still lack knowledge about the symptoms of most diseases in groundnut. Training farmers on disease management will help them to develop local strategies to face some of these constraints.

## Conclusion

The study provided recent information on groundnut production in Burkina, which is characterized by an extensive cropping system in an environment largely dominated by cereals and in a subsistence agriculture system. It was evident that women are highly involved in groundnut production but with limited and inequitable access to production resources. The crop is found to be constrained by several abiotic and biotic factors that impacted its production and constitute a bottleneck for the groundnut sector. This situation is worsened by an unstructured and unorganized groundnut sector and a weak breeding program and a seed production and distribution system. Finding a way to overcome these constraints constitute a primary step to alleviate groundnut farmer conditions and enhance groundnut production in Burkina Faso. Therefore, there is a need for new technologies and strategies to tackle and enhance groundnut production in Burkina Faso. These include strengthening the breeding program, seed production companies, and extension services for enhanced generation and dissemination of technologies. Breeding of new varieties must take into account the specific farmers’ preference and market needs of target areas for better adoption of these varieties.

## Data Availability

All data generated or analyzed during this study are included in this published article.
